# LPA_1_ signaling drives Schwann cell dedifferentiation in experimental autoimmune neuritis

**DOI:** 10.1186/s12974-021-02350-5

**Published:** 2021-12-17

**Authors:** Fabian Szepanowski, Maximilian Winkelhausen, Rebecca D. Steubing, Anne K. Mausberg, Christoph Kleinschnitz, Mark Stettner

**Affiliations:** Department of Neurology and Center for Translational Neuro- and Behavioral Sciences (C-TNBS), University Medicine Essen, University Duisburg-Essen, Hufelandstr. 55, 45147 Essen, Germany

**Keywords:** Lysophosphatidic acid (LPA), Experimental autoimmune neuritis (EAN), Inflammatory neuropathy, Guillain–Barré-syndrome, Schwann cell, Differentiation, Demyelination, Remyelination, Sox2, Sox10

## Abstract

**Background:**

Lysophosphatidic acid (LPA) is a pleiotropic lipid messenger that addresses at least six specific G-protein coupled receptors. Accumulating evidence indicates a significant involvement of LPA in immune cell regulation as well as Schwann cell physiology, with potential relevance for the pathophysiology of peripheral neuroinflammation. However, the role of LPA signaling in inflammatory neuropathies has remained completely undefined. Given the broad expression of LPA receptors on both Schwann cells and cells of the innate and adaptive immune system, we hypothesized that inhibition of LPA signaling may ameliorate the course of disease in experimental autoimmune neuritis (EAN).

**Methods:**

We induced active EAN by inoculation of myelin protein 2 peptide (P2_55–78_) in female Lewis rats. Animals received the orally available LPA receptor antagonist AM095, specifically targeting the LPA_1_ receptor subtype. AM095 was administered daily via oral gavage in a therapeutic regimen from 10 until 28 days post-immunization (dpi). Analyses were based on clinical testing, hemogram profiles, immunohistochemistry and morphometric assessment of myelination.

**Results:**

Lewis rats treated with AM095 displayed a significant improvement in clinical scores, most notably during the remission phase. Cellular infiltration of sciatic nerve was only discretely affected by AM095. Hemogram profiles indicated no impact on circulating leukocytes. However, sciatic nerve immunohistochemistry revealed a reduction in the number of Schwann cells expressing the dedifferentiation marker Sox2 paralleled by a corresponding increase in differentiating Sox10-positive Schwann cells. In line with this, morphometric analysis of sciatic nerve semi-thin sections identified a significant increase in large-caliber myelinated axons at 28 dpi. Myelin thickness was unaffected by AM095.

**Conclusion:**

Thus, LPA_1_ signaling may present a novel therapeutic target for the treatment of inflammatory neuropathies, potentially affecting regenerative responses in the peripheral nerve by modulating Schwann cell differentiation.

## Introduction

Immune-mediated neuropathies represent a heterogeneous group of rare peripheral nerve disorders comprising both acute and chronic forms. The prototype of an acute monophasic inflammatory polyneuropathy is the Guillain–Barré syndrome (GBS), with a reported incidence rate of 1–4 cases per 100,000 worldwide [1]. The prevalence of chronic inflammatory demyelinating polyneuropathy (CIDP) is estimated to be 1–2 of 100,000 individuals [2].

While GBS and CIDP are distinct disorders, they share clinical and pathogenetic aspects: immunomodulatory therapies such as plasma exchange or intravenous immunoglobulins (IVIg) represent effective treatment options in both conditions. Moreover, the presence of cellular infiltrates consisting mainly of T-lymphocytes and macrophages in nerve biopsies of GBS and CIDP patients as well as in the respective animal models, further supports the idea of an immune-mediated pathogenesis for either condition [3, 4].

Given the substantial body of evidence indicating autoimmune-driven damage to neurons and glial cells as major pathological hallmark of inflammatory neuropathies, cellular and humoral immune responses against the peripheral nervous system (PNS) have mostly been considered in isolation, largely ignoring the acute and potentially long-lasting impact on physiological homeostasis in the peripheral nerve. While the detrimental effects of innate and adaptive immune responses have been well-defined in autoimmune neuropathies [5], demyelination does not appear to be an exclusive result of self-directed immunity, but rather a result of Schwann cell dedifferentiation and thus the adoption of a non-myelinating phenotype in the inflammatory milieu via the secretion of cytokines and various other cues from T-lymphocytes and their effector cells [6].

Schwann cells, as the myelin-forming glial cell population of the PNS, have long been considered as passive bystanders in peripheral neuroinflammation. While Schwann cells have clearly been shown to fulfill important functions beyond building myelin sheaths [7], such as providing trophic and structural support to axons, several lines of evidence further support a view that strongly indicates an enormous phenotypic plasticity of Schwann cells. Indeed, Schwann cells are increasingly accepted to be capable of responding to a wide range of stimuli and appear as facultative immunocompetent cells that may even actively orchestrate inflammatory processes in the PNS, functionally resembling innate immune cells such as macrophages [8–10].

A recent study took the effort to analyze Schwann cell differentiation in NOD/B7-2-knockout mice, a spontaneous inflammatory demyelinating neuropathy model that shares several pathological features with CIDP. It was demonstrated that the onset of neuroinflammation resulted in an upregulation of dedifferentiation-associated genes. Most notably, the downregulation of Krox20, the master regulator of peripheral myelination, was paralleled by the induction of c-Jun expression, a dedifferentiation marker [11]. These findings appear particularly revealing in light of a pilot immunohistochemical study that investigated the expression of c-Jun in nerve and skin biopsies from neuropathy patients. Whereas c-Jun expression was barely detectable in nerves of healthy controls, nerves from patients with different neuropathies, including GBS and CIDP, displayed notable immunoreactivity mostly confined to Schwann cells [12]. Together, these studies point to Schwann cell dedifferentiation as a central mechanism of demyelination in inflammatory neuropathies beyond mere cellular or myelin damage.

Lysophosphatidic acid (LPA) is a pleiotropic lipid messenger that addresses at least six specific G-protein-coupled LPA receptors [13]. While LPA and its receptors fulfill critical functions in the developing vascular, nervous and immune system, LPA also plays significant pathophysiological roles in various diseases, including systemic sclerosis and idiopathic pulmonary fibrosis. For these conditions, LPA receptor antagonists are in clinical development [13, 14]. Interestingly, LPA signaling has also been associated with the development of neuropathic pain and peripheral nerve demyelination [14–16]. The demyelinating effect of LPA appears to be predominantly mediated through the LPA_1_receptor expressed on Schwann cells, contributing to the inverse regulation of the positive and negative differentiation factors Sox10 and Sox2 [17, 18]. Moreover, LPA might be involved in inflammatory cytokine release from both glial and myeloid cells as well as various other cell types [13, 17, 19].

Given the potential impact of LPA_1_ signaling on myelination in addition to a possible aggravation of inflammatory responses, we hypothesized that LPA signaling might be involved in the pathophysiology of inflammatory neuropathies. To explore this hypothesis, experimental autoimmune neuritis (EAN), the animal model of GBS, was induced in Lewis rats and animals were treated with the specific orally available LPA_1_ receptor antagonist AM095. Here, we report on the potential role of LPA in Schwann cell dedifferentiation associated with inflammatory neuropathies.

## Materials and methods

### Induction of experimental autoimmune neuritis (EAN)

For all experiments, female Lewis rats aged 8–12 weeks were used. Animals were bred and housed under specific pathogen-free conditions at the animal research facility of the University Medicine Essen. To induce active EAN, rats received subcutaneous (footpad) injections of 200 µg P2_55–78_ (JPT peptides, Berlin, Germany) as previously described [20]. The following EAN score was applied: 0 no impairment, 1 reduced tail tone, 2 limp tail, 3 absent righting reflex, 4 gait abnormalities, 5 mild paraparesis, 6 moderate paraparesis, 7 severe paraparesis or paraplegia, 8 tetraparesis, 9 moribund, and 10 death due to neuropathy.

### Treatment with AM095

The LPA_1_ receptor antagonist AM095 [21] was obtained from ApexBio (Houston, TX, USA) and dissolved in phosphate buffered saline (PBS) containing 0.5% methylcellulose (Sigma-Aldrich). Treatment was initiated with development of first clinical signs of EAN approximately 10 dpi. From this point onwards, animals received AM095 at a concentration of 10 mg/kg body weight once daily via oral gavage. Controls received an equal volume of vehicle.

### Tissue preparation for immunohistochemistry

At the indicated time points (14, 21 and 28 dpi), animals were killed and perfused with PBS before sciatic nerves were sampled. Nerves were carefully taken by only handling the proximal end (approx. 1 cm from distal branching) with forceps and cutting the most distal end using scissors. Nerves were placed in cryomolds, covered with cryo-embedding compound and placed on dry ice. Samples were stored at − 80 °C. Longitudinal sections were prepared at a thickness of 7 µm in a cryostat chamber and sections were stored at − 20 °C before further processing.

### Hemogram profile

At 21 dpi, immediately following animal sacrifice, blood was drawn via cardiac puncture using heparinized cannulae. Blood samples were collected in tubes containing EDTA and were carefully mixed to avoid clotting. The ProCyte Dx hematology analyzer (IDEXX, USA) was used for the generation of a complete hemogram.

### Immunohistochemistry

All sections were post-fixed in 4% paraformaldehyde for 20 min. Immunohistochemical procedures were then performed as described previously [22].

Briefly, slides were washed once PBS and twice in PBT (PBS + 0.1% Triton X-100) for 5 min each. Slides were incubated with blocking solution (10% normal goat serum in PBT) for 30 min at room temperature. Primary antibody was applied and slides were incubated at 4 °C overnight. Slides were washed twice for 5 min in PBT, and secondary antibody was applied and incubated at room temperature for 1 h. Slides were washed 5 min in PBT and 5 min in PBS and mounted with Mowiol containing 4,6′diamidino-2-phenylindole (DAPI). Images were acquired using a Leica DMi8 microscope.

### Antibodies

The following antibodies were used at the indicated dilutions: rabbit monoclonal [SP7] anti-CD3 (abcam; diluted 1:500); mouse monoclonal [1F4] anti-CD3 (BioLegend; diluted 1:1000); mouse monoclonal [OX42] anti-CD11b/c (abcam; diluted 1:1000); rabbit polyclonal anti-Sox2 IgG (abcam, ab97959; diluted 1:200); rabbit monoclonal [EPR4007] anti-Sox10 (abcam; diluted 1:250).

### Preparation of toluidine blue-stained semi-thin sections

For the preparation of semi-thin sections, sciatic nerves were fixed in 0.1 M cacodylate buffer containing 2.5% glutaraldehyde for 24 h at 4 °C. Further processing and staining procedures were performed as described previously [23]. Briefly, nerves were washed in cacodylate buffer followed by incubation in an osmium tetroxide reagent for 3 h. Osmium tetroxide reagent was composed of one part 5% potassium dichromate solution (pH 7.4), one part 3.4% NaCl solution, and two parts 2% osmium tetroxide. Samples were dehydrated in an ascending ethanol series. Following dehydration, samples were incubated in propylene oxide in tightly closed containers for 1 h at room temperature, then 1 h in a 1:1 mixture of propylene oxide/epon (epoxy embedding medium kit; Sigma-Aldrich), and finally kept at 4 °C in epon only overnight. Samples were placed in silicone molds and covered with epon embedding mixture. Embedded samples were incubated at 37 °C for 6 h, at 47 °C for 15 h, and finally at 60 °C for 28 h until epon was completely hardened. Transverse sections were prepared at a thickness of 1 μm at an ultracut microtome and immediately stained with 1% toluidine blue solution and mounted in xylene-based medium.

### Assessment of morphometric data

Morphometric analysis was performed by a blinded investigator using ImageJ (National Institutes of Health, Bethesda, MA, USA). For the evaluation of g-ratios and axonal diameters, the circumference of axons and their respective myelin sheaths was measured within randomly selected fields. A minimum of 200 axons per nerve was evaluated. For the calculation of g-ratios, axonal circumference was divided by the circumference of the respective myelin sheath.

### Image and data analysis

Analysis of images was performed using ImageJ (National Institutes of Health, Bethesda, MA, USA). For the quantification of cell populations, three longitudinal sections per animal were photographed with a high magnification (Leica DMi8). Cells were counted manually by a blinded investigator using ImageJ and either adjusted to the analyzed area or normalized to total cell count of DAPI-positive cells, respectively. Data analysis and compilation of graphs was performed using Microsoft (Redmond, WA, USA) Excel and GraphPad (La Jolla, CA, USA) Prism 7. Statistical analysis was conducted using Student’s *t*-test; multiple comparisons were performed by multiple *t*-tests corrected by the Holm–Sidak method. Non-parametric data were analyzed by Mann–Whitney test. Statistical significance is indicated by asterisks with *P* ≤ 0.05*, *P* ≤ 0.01**, and *P* ≤ 0.001***.

## Results

### AM095 accelerates recovery in active EAN by clinical and morphometric measures

**Fig. 1:**
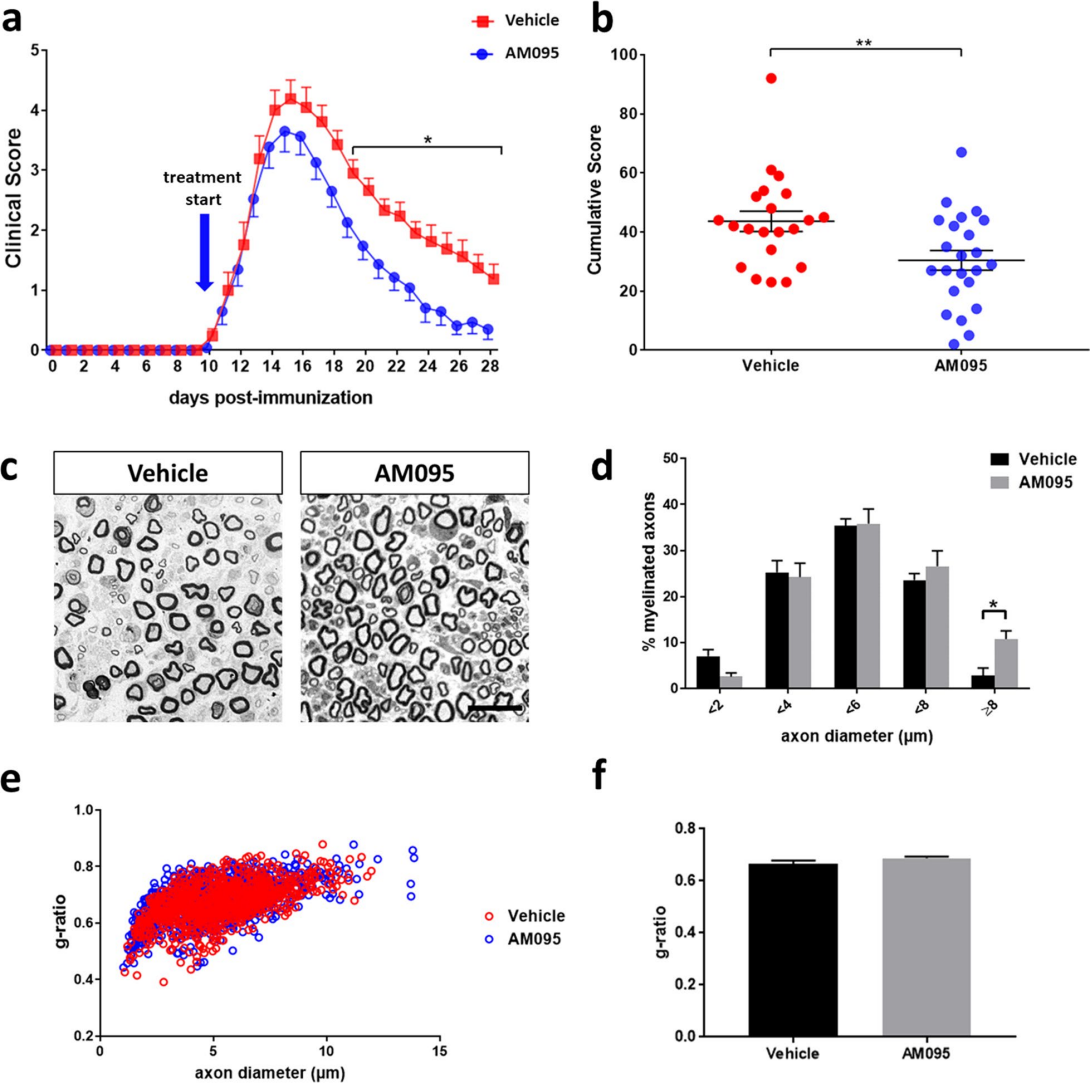
Inhibition of LPA_1_ mitigates disease severity in EAN. **a** Active EAN was induced in Lewis rats, which were treated with the LPA_1_ receptor antagonist AM095 once daily after developing first clinical symptoms at 10 dpi. While there was no significant impact of AM095 around the peak of disease, disease severity was significantly ameliorated over the course of the remission phase. A significant difference was observed from 20 dpi until the end of the experiment at 28 dpi. Each dot indicates mean clinical scores ± s.e.m. **b** Cumulative clinical scores of individual animals obtained from day 0 to the end of the experiment at 28 dpi. Animals were pooled from three independent experiments. *N* = 21 (Vehicle)/23 (AM095). **c** Representative images of semi-thin toluidine blue-stained sections of sciatic nerves at 28 dpi. **d** Axon diameter histogram indicates a significant increase in the number of large-caliber (≥ 8 µm) myelinated axons following AM095 treatment (*N* = 4). **e** G-ratios were plotted against axon diameters; overlapping results indicate comparability of both treatment groups with regard to axon diameter distributions and myelination. **f** In accordance with this finding, the assessment of g-ratios (the numerical ratio between axonal and myelinated fiber diameter) does not indicate any differences with regard to myelin thickness (*N* = 4). Data represent mean ± s.e.m. *P* ≤ 0.05*. Scale bar indicates 50 µm

Following the induction of active EAN, rats were monitored daily and clinical scores were determined until 28 days post-immunization (dpi). The animals were treated with the specific LPA_1_ antagonist AM095 at 10 mg/kg via oral gavage once daily in a therapeutic regime, starting with the onset of first clinical signs of EAN, approximately 10 dpi. No effect of AM095 on disease severity was noted up to the peak of the disease. However, a significant improvement and markedly accelerated recovery was observed in the AM095-treated versus vehicle group shortly after the beginning of the remission phase from 20 dpi onwards up to the end of the experiment at 28 dpi (Fig. 1a). Accordingly, cumulative clinical scores were significantly improved under AM095 (Fig. 1b). To further substantiate these clinical findings with histological measures, morphometric analyses of toluidine blue-stained semi-thin sections were performed at 28 dpi. In line with the clinical improvement, a significantly increased proportion of large-caliber myelinated axons was identified following AM095 treatment (Fig. 1c, d), while there was no impact of AM095 on overall myelin thickness as determined by g-ratio measurements (Fig. 1e, f).

### AM095 shows only a minor impact on immune cell infiltration

**Fig. 2:**
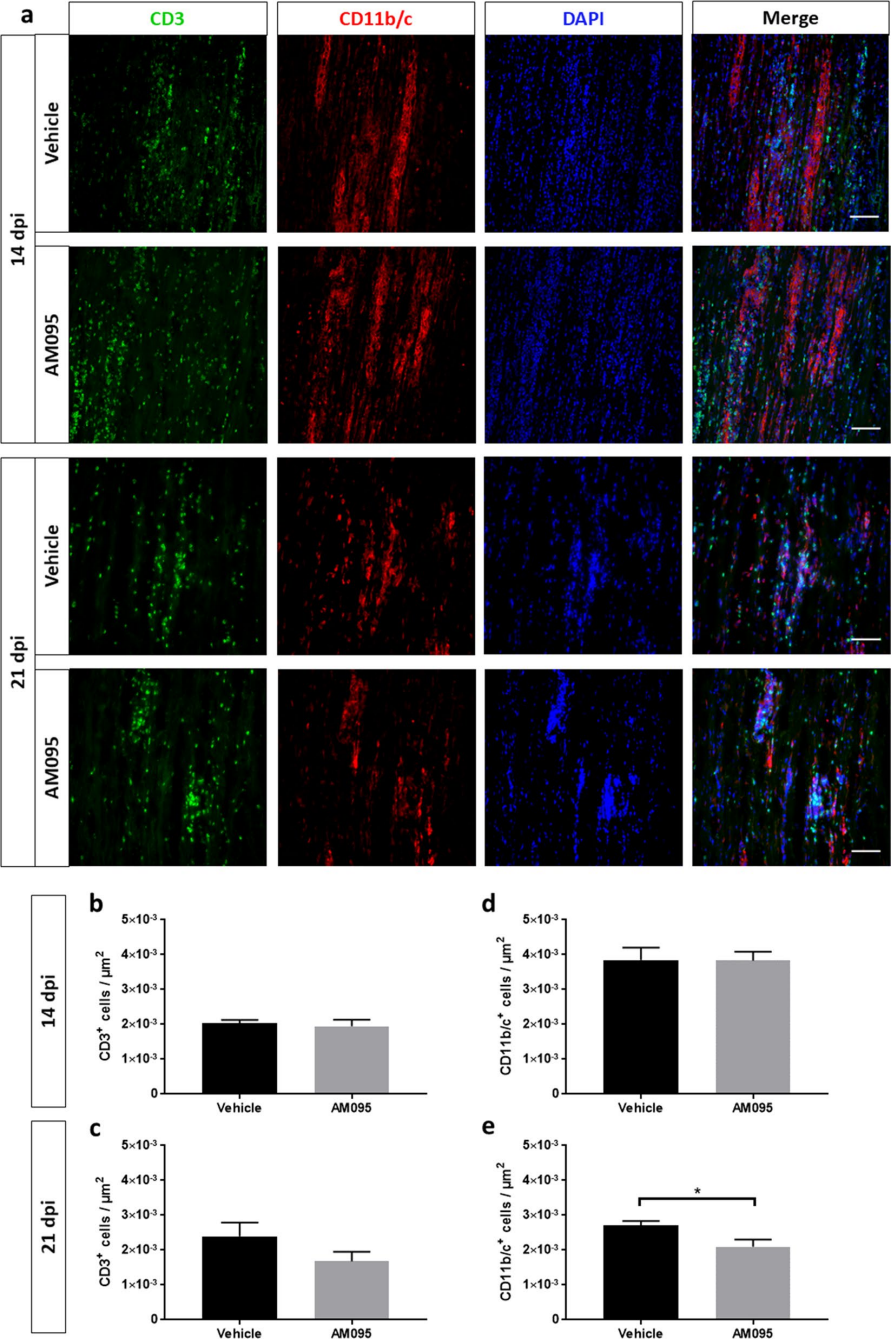
Minor impact of AM095 treatment on immune cell infiltration. Immune cell infiltration was assessed by counting the number of CD3^+^ (T-lymphocytes) and CD11b/c^+^ (myeloid cells) on sciatic nerve sections in randomly selected fields. **a** Representative images of immune cell infiltration at peak of disease at 14 dpi, and early remission phase at 21 dpi. **b** + **c** No significant difference was observed in the number of infiltrating CD3+ cells between peak of disease and remission phase (*N* = 9/8/4/4 from left to right). **d** + **e** While there was no significant reduction in CD3^+^ cells at 14 dpi, we recognized a slight decrease in myeloid cells under AM095 at 21 dpi (*N* = 5/5/5/6 from left to right)). Cell counts were normalized to area. Data represent mean ± s.e.m. *P* ≤ 0.05*. Scale bars indicate 50 µm

**Fig. 3:**
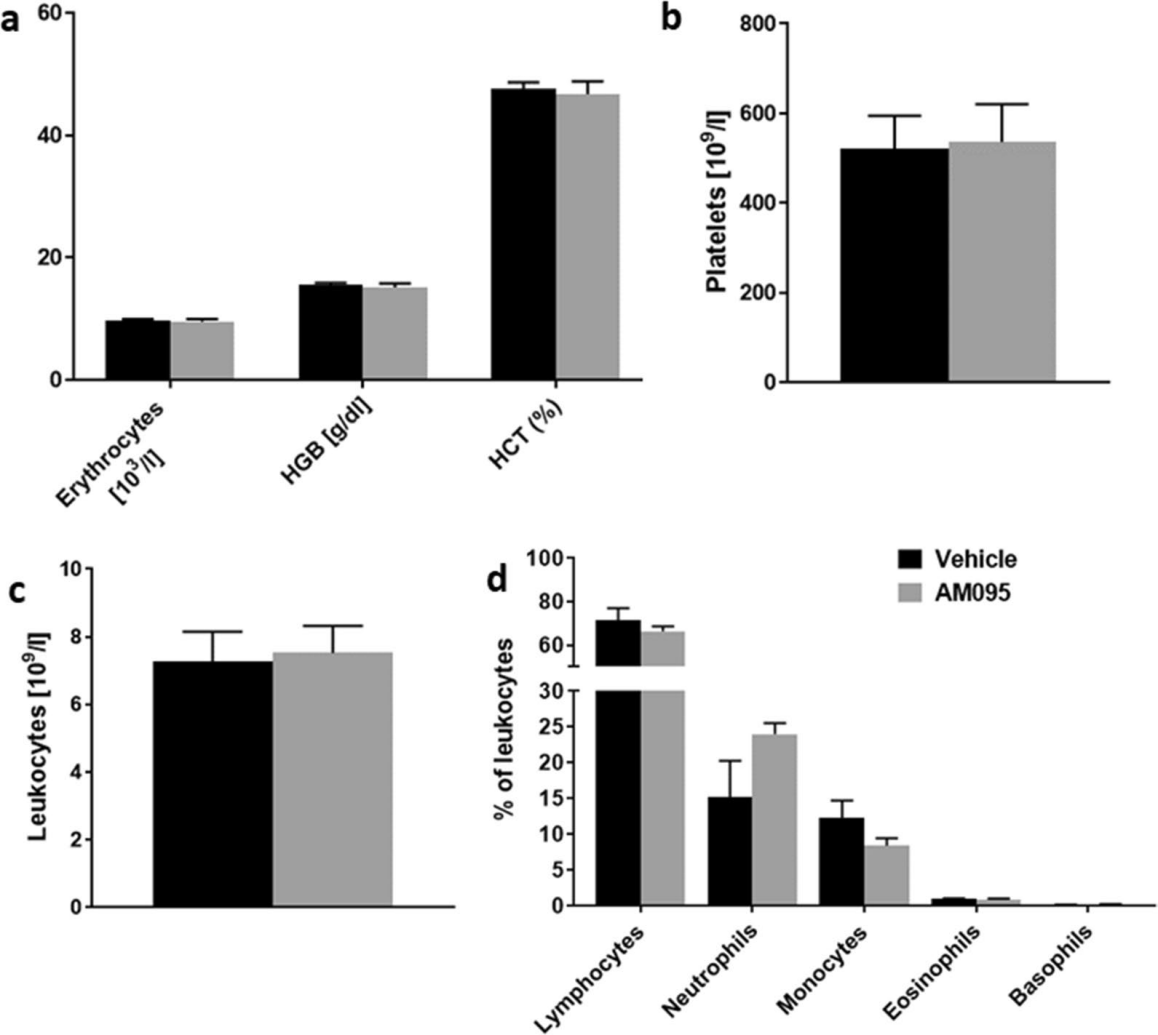
Hemogram profiles indicate no impact of AM095 a circulating leukocytes. At 21 dpi, hemogram profiles were generated from blood samples taken by cardiac puncture immediately after animals were sacrificed. No differences were detected in basic parameters including **a** erythrocytes, hemoglobin (HGB), hematocrit (HCT) and **b** platelets. **c** The total number of circulating leukocytes was unaffected by treatment with AM095. **d** Coherently, no significant differences were observed for subpopulations including lymphocytes, neutrophils, monocytes, eosinophils and basophils. Data represent mean ± s.e.m. *N* = 7 for each column

The potential for AM095 to reduce inflammatory infiltrates was assessed by examination of the numbers of CD3^+^ T-lymphocytes as well as CD11b/c^+^ myeloid cells at the peak of disease (14 dpi) and during remission phase (21 dpi) on sciatic nerve sections by immunohistochemistry. At the peak of disease (14 dpi), neither the number of T-lymphocytes nor myeloid cells were changed by AM095 (Fig. 2b, d). At the beginning of the remission phase (21 dpi), a significant reduction in the number of CD11b/c^+^ cells and a mean, however, non-significant reduction in CD3^+^ cells was observed, possibly indicating a slightly accelerated clearance of immune cells from the sciatic nerve (Fig. 2c, e). Moreover, at 21 dpi, we generated hemogram profiles, indicating no overall impact of AM095 on circulating leukocytes (Fig. 3).

### AM095 increases the number of redifferentiating Schwann cells

**Fig. 4:**
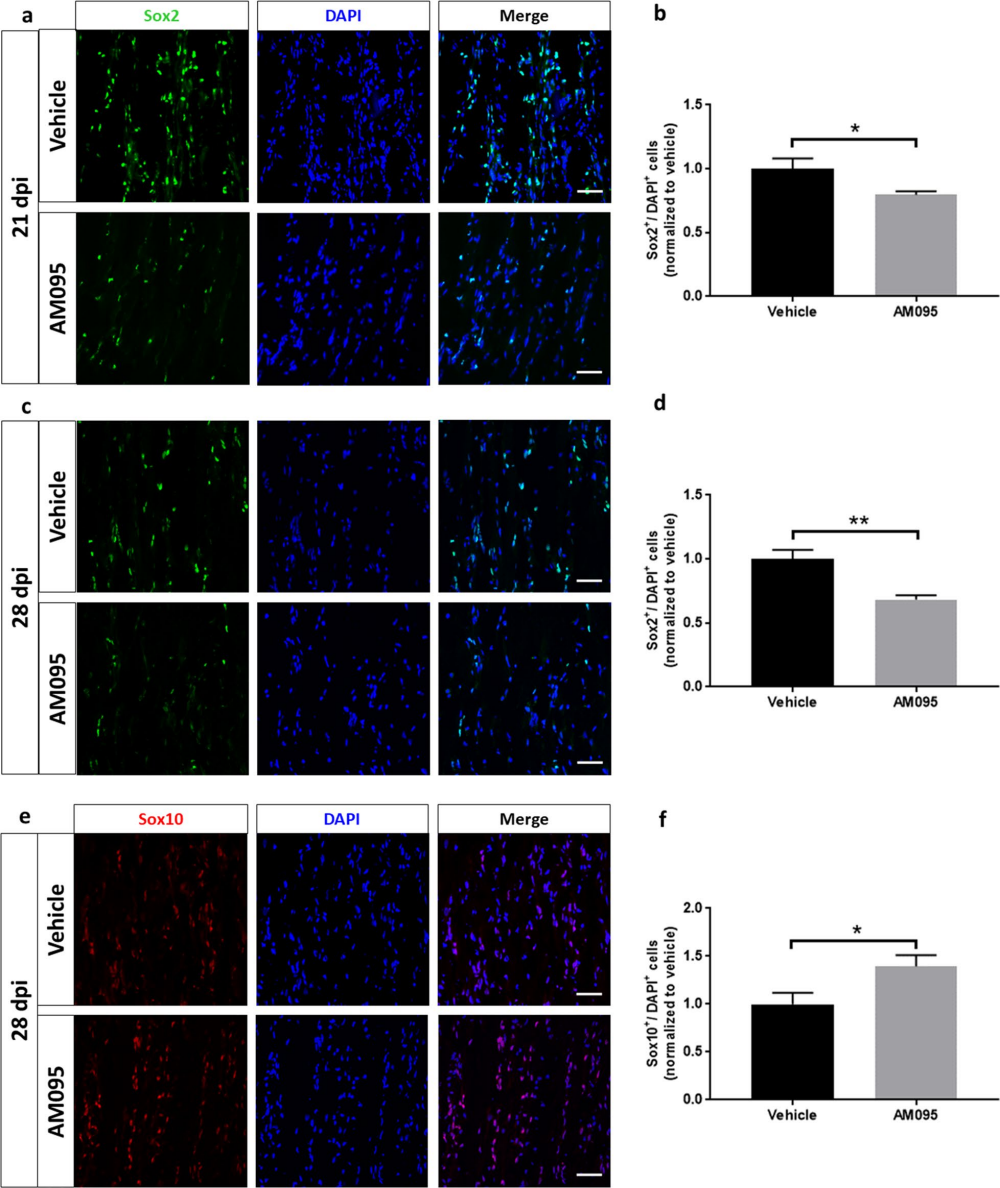
AM095 treatment decreases the number of dedifferentiating Schwann cells. Schwann cell dedifferentiation was investigated via immunohistochemistry by counting the number Sox2-positive cells in randomly selected fields on sciatic nerve sections. **a** + **b** A significant reduction of Sox2-positive cells with AM095 was observed at 21 dpi (*N* = 5/5). **c** + **d** This effect was even more pronounced at 28 dpi (*N* = 8/8). **e** + **f** In accordance with this finding, the number of differentiated Sox10-positive cells was significantly increased under treatment with AM095 at 28 dpi (*N* = 8/8). The number of Sox2/10-positive cells was related to the total number of cells stained with DAPI and normalized to vehicle-treated controls. Data represent normalized mean ± s.e.m. *P* ≤ 0.05*, *P* ≤ 0.01**. Scale bars indicate 50 µm

As a potential mechanism underlying the clinical improvement observed at the beginning of the remission phase, the expression of Schwann cell differentiation markers was examined. At 21 dpi, a substantial number of Schwann cells was found to express the dedifferentiation marker Sox2 (Fig. 4a, b). The number of Sox2-positive cells was significantly reduced following treatment with AM095. This effect was even more pronounced at 28 dpi (Fig. 4c, d). In line with this, a corresponding significant increase in the number of cells expressing the positive differentiation marker Sox10 was observed at 28 dpi (Fig. 4e, f).

**Fig. 5:**
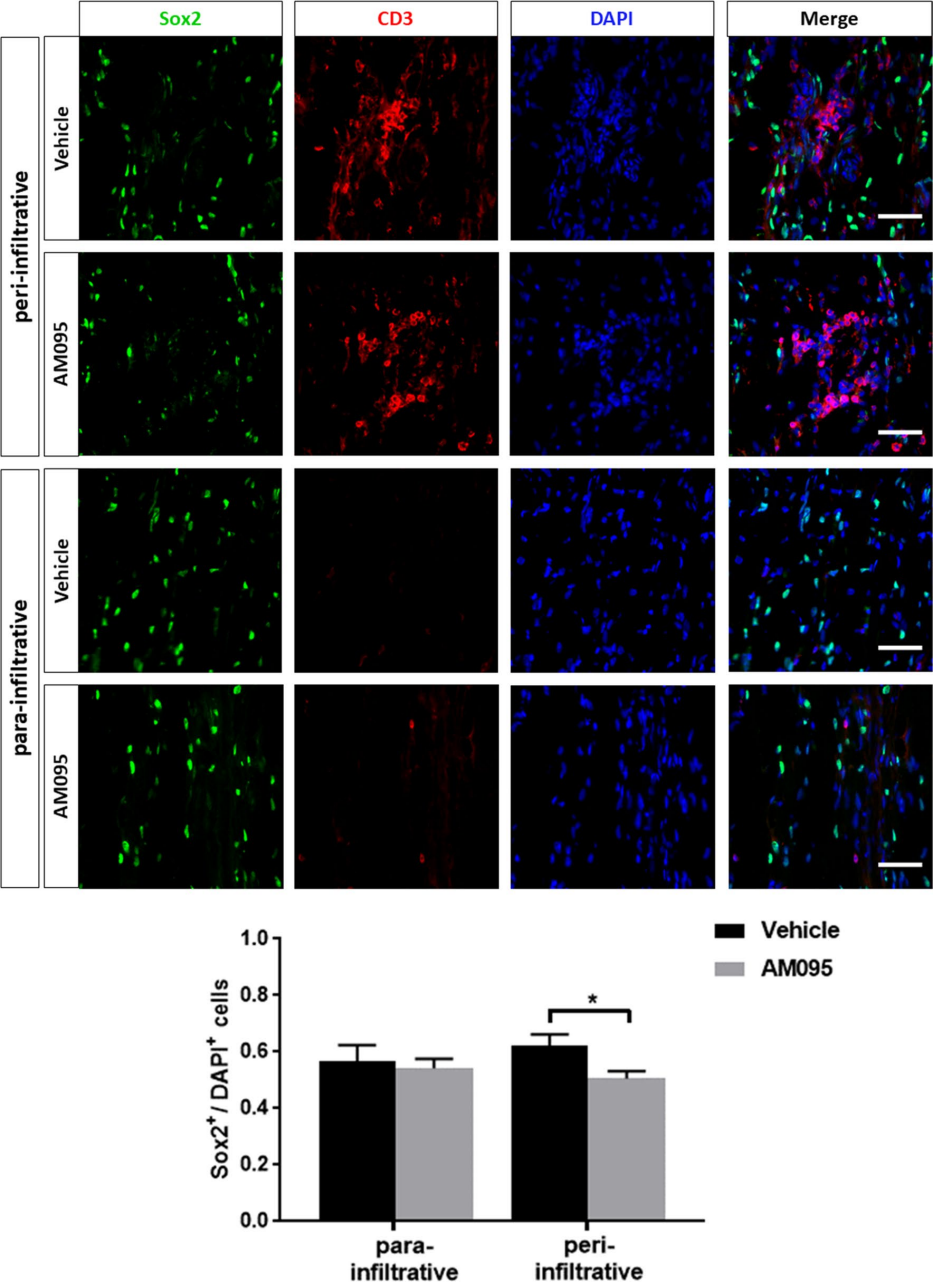
Proximity to immune cell infiltrates may foster Schwann cell dedifferentiation. Immunohistochemical analysis of Sox2-positive cells in relation to cellular infiltrates was conducted. Sox-2-positive cells were counted in close proximity to cellular infiltrates (termed “peri-infiltrative”) as well as in areas without marked infiltration (termed “para-infiltrative”). Cellular infiltrates were defined as an accumulation of DAPI-positive cells and positivity for CD3 staining. “Peri-infiltrative” was defined as the area surrounding an infiltrate by twice the circumference of that respective infiltrate. The number of Sox2-positive cells was related the number of DAPI-positive cells. Data represent mean ± s.e.m. *N* = 4 for each column. *P* ≤ 0.05*. Scale bars indicate 50 µm

Interestingly, we noted that Schwann cells appeared to preferentially dedifferentiate in closer proximity to inflammatory infiltrates. Therefore, at 21 dpi, cells expressing the dedifferentiation marker Sox2 were counted in areas closely surrounding cellular infiltrates (termed “peri-infiltrative”) and in areas with greater distance to infiltrates (termed “para-infiltrative”) (Fig. 5). Infiltrates were identified by an accumulation of DAPI^+^ cells and positivity for CD3 staining, “peri-infiltrative” was defined as the area surrounding an infiltrate by approximately twice the circumference of that respective infiltrate. No significant difference was detected in the number of Sox2^+^ cells in para-infiltrative spaces, however, a reduction in Sox2^+^ cells following AM095 treatment was detected in peri-infiltrative areas (Fig. 5), indicative of an interaction between immune and Schwann cells in the process of Schwann cell dedifferentiation.

## Discussion

In the past decade, enormous progress has been made in understanding the regulation of Schwann cell differentiation dynamics, both in developmental and pathophysiological contexts. Several positive and negative regulators of Schwann cell differentiation have been identified. On the transcriptional level, the most prominent positive differentiation factors include Oct6 and Sox10, concertedly inducing expression of the master regulator of peripheral myelination, Krox20 [24–26]. Negative regulators such as c-Jun or Sox2 are typically activated in the event of nerve injury, promoting the transition from a myelinating phenotype to one that propagates Wallerian degeneration, initially characterized by the secretion of inflammatory mediators and induction of phagocytic activity [27–31]. Over the course of nerve regeneration, Schwann cells adopt a repair phenotype, providing guidance and trophic support to regenerating axons in Bungner’s bands. Negative regulators of myelination such as c-Jun or Sox2 also play a crucial role in the adaption of this repair phenotype [32]. As to whether these factors and the process of Schwann cell dedifferentiation contribute to demyelination in inflammatory neuropathies has been rarely explored. One pilot study identified c-Jun expressing Schwann cells in various types of neuropathies including GBS and CIDP [12]. An upregulation of c-Jun was also reported in parallel to the onset of neuroinflammation in the NOD/B7-2-knockout mouse model of chronic neuritis [11]. Pro-inflammatory cytokines involved in inflammatory neuropathies, such as interleukin-17, have been demonstrated to induce Schwann cell dedifferentiation even in the absence of a direct immune cell interaction [33]. Although LPA is not classified as an inflammatory cytokine, it is known to be secreted in various tissues and cell types and might aggravate immune reactions by promoting the release of inflammatory mediators such as cytokines and matrix metalloproteinases [13, 34]. In the context of peripheral nerve injury, it was previously demonstrated that LPA significantly contributes to dedifferentiation and inflammatory activation of Schwann cells. Most notably, antagonism of LPA_1_ using AM095 reduced the expression of the negative regulator Sox2 while increasing the expression of Sox10 in this murine model of nerve injury. Moreover, this effect was associated with reduced demyelination in response to LPA treatment in murine DRG cultures [17]. Consistent with these findings, LPA has been shown to induce downregulation of Sox10 and subsequently myelin proteins such as myelin basic protein (MBP) and myelin protein zero (P0) in dependence on acetylation of NFκB [18]. Acetylation of NFκB has been implicated in the inhibition of myelin-associated gene expression including Sox10, Oct6 and Krox20 [35].

Interestingly, in the study presented herein, amelioration of clinical scores following AM095 treatment was statistically significant only from 20 dpi onwards. This finding suggests that LPA_1_ antagonism might predominantly affect regenerative responses in the peripheral nerve rather than modulating inflammatory processes. Therefore, administration of AM095 might be of particular interest in a therapeutic rather than a preventative context. To assess the impact of AM095 on nerve regeneration, semi-thin sections of sciatic nerves were examined at 28 dpi to determine morphometric parameters. While there was no overall impact of AM095 on myelin thickness and axonal size distribution, we detected a significant increase in large-caliber myelinated axons under AM095 treatment, further strengthening the argumentation for regenerative effects of the compound.

T-lymphocyte chemotaxis and matrix metalloproteinase secretion have been shown to be stimulated by LPA_2_ receptor signaling, but not LPA_1_ [36]. As such, AM095 would appear rather unlikely to affect T-lymphocyte migration. In line with this hypothesis, no significant difference was detected with regard to infiltrating CD3^+^ T-lymphocytes or CD11b/c^+^ myeloid cells at the peak of disease. Limited evidence pointed to a slightly accelerated immune cell clearance at the beginning of the remission phase following AM095 treatment. However, there was no impact on the number of circulating immune cells. Based on our clinical, morphometric and immunohistochemical findings, these data strongly indicate that LPA_1_ antagonism might predominantly modulate nerve regeneration during the remission phase.

To better understand the mechanism by which AM095 accelerated recovery, markers for Schwann cell differentiation were explored using immunohistochemical detection of the aforementioned negative and positive differentiation factors Sox2 and Sox10 on longitudinal sciatic nerve sections obtained from rats over the course of the remission phase at 21 and 28 dpi. At both time points, the number of Sox2-expressing cells was significantly decreased under AM095 treatment, and this effect was even more pronounced at 28 dpi. This reduction in Sox2-positive cells was paralleled by a significant increase of Sox10 expressing cells at 28 dpi, pointing to a greater number of redifferentiating Schwann cells at this stage. Notably, a greater proportion of Schwann cells expressed Sox2 in closer proximity to cellular infiltrates with CD3 positivity. This finding suggests that immune cells might foster Schwann cell dedifferentiation via LPA in a paracrine manner. The function of LPA as a pro-inflammatory paracrine messenger has already been extensively illustrated in inflammatory conditions such as cancer-related or airway inflammation and rheumatoid arthritis [34]. In this regard, LPA might fulfill a central role in the propagation of inflammation-driven Schwann cell dedifferentiation.

## Conclusion

Collectively, these findings indicate that a promotion of Schwann cell redifferentiation might be a relevant therapeutic strategy in inflammatory neuropathies both by clinical and morphometric measures and that modulating LPA signaling using selective LPA_1_ antagonism may provide a potential therapeutic target to block inflammatory glial cell activation and propagate remyelination in autoimmune neuropathies.

## Data Availability

All methods and data supporting the conclusions of this work have been included in the article.

## Abbreviations

LPA: Lysophosphatidic acid
EAN: Experimental autoimmune neuritis
dpi: Days post-immunization
GBS: Guillain–Barré syndrome
CIDP: Chronic inflammatory demyelinating polyneuropathy
PNS: Peripheral nervous system
NOD: Non-obese diabetic
MBP: Myelin basic protein
P0: Myelin protein zero
P2: Peripheral myelin protein 2
PBS: Phosphate buffered saline
